# Exogenous Melatonin Confers Salt-Alkali Tolerance in *Fraxinus mandshurica* by Orchestrating Resource Allocation and Activating Phenylpropanoid-Mediated Defenses

**DOI:** 10.3390/plants15030438

**Published:** 2026-01-30

**Authors:** Junqi Yu, Ziye Xu, Fan Huang, Jingqi Yin, Wenqian Dai, Yinglun Sun, Chi Zhang, Tongbao Qu

**Affiliations:** College of Forestry and Grassland Science, Jilin Agricultural University, Changchun 130024, China; 15648088896@163.com (J.Y.); eyzixu@163.com (Z.X.); 18365105429@163.com (F.H.); 13079726997@163.com (J.Y.); 13944947317@163.com (W.D.); sunyinglun123@163.com (Y.S.); 18686610130@163.com (C.Z.)

**Keywords:** melatonin, *Fraxinus mandshurica*, salt stress, transcriptomics, metabolomics

## Abstract

The physiological mechanism of melatonin in alleviating combined saline-alkali stress in *Fraxinus mandshurica* remains unclear. This study aimed to determine the efficacy of exogenous melatonin in enhancing salt tolerance and elucidate the underlying mechanisms through integrated physiological and multi-omics analyses. Seedlings were subjected to 400 mmol L^−1^ saline-alkali stress and treated with foliar melatonin. We quantified key growth indicators (height, diameter, dry biomass) and measured the activities of antioxidant enzymes (SOD, POD). Melatonin significantly alleviated growth inhibition, increasing biomass and height by 29% and 13%, respectively, while enhancing net photosynthetic rate and antioxidant capacity. To uncover the systemic regulation, conjoint analysis of transcriptome (RNA-seq) and metabolome data was performed. This integrated approach revealed that melatonin specifically activated common KEGG pathways pivotal for stress adaptation, including plant hormone signal transduction, phenylpropanoid biosynthesis, and starch and sucrose metabolism, with coordinated upregulation of associated genes and metabolites. Collectively, our integrated data demonstrate that melatonin enhances *Fraxinus* tolerance by synergistically improving photosynthesis and antioxidant defense, underpinned by a reconfigured molecular network. This study provides a theoretical basis for using melatonin as an eco-friendly biostimulant to improve woody plant resilience in saline-alkali soils.

## 1. Introduction

Salt stress is a major abiotic stress limiting crop and forest productivity [1,2], characterized by its extensive impact and prolonged duration. Most *Fraxinus* species (especially *Fraxinus nigra*) exhibit moderate to high sensitivity to salinity, severely restricting their application and distribution in saline-alkali areas [3,4]. Specifically, salinity induces severe oxidative damage and impairs photosynthetic function in this genus [5,6]. Thus, enhancing their salt tolerance is an urgent practical challenge.

Physiologically, salt stress inhibits the growth and photosynthetic performance of *Fraxinus* species, largely via oxidative damage [7,8]. Morphologically, alterations in root architecture under salinity may further compromise water and nutrient uptake. However, a systematic understanding of how these physiological and morphological responses are integrated in *F. mandshurica*—particularly under combined saline-alkali conditions—is still lacking. This knowledge gap limits the development of targeted mitigation strategies for this valuable species.

Traditional approaches face numerous limitations: genetic improvement cycles are lengthy, and chemical amendments pose environmental risks [9,10]. Biostimulants offer an environmentally friendly alternative strategy [11]. Among numerous candidate substances, melatonin—as an endogenous signaling molecule—has been demonstrated to effectively alleviate multiple abiotic stresses in herbaceous plants [12,13,14]. For example, in tomatoes (*Solanum lycopersicum* L.), overexpressing the melatonin synthesis gene *SlCOMT1* to elevate endogenous melatonin levels significantly alleviates salt stress-induced oxidative damage, thereby enhancing salt tolerance. Similarly, exogenous melatonin treatment effectively promotes germination and growth of cucumber (*Cucumis sativus*) seedlings under salt stress by regulating the biosynthesis of abscisic acid (ABA) and gibberellin (GA). These effects are further elucidated in rice (*Oryza sativa* L.): melatonin alleviates ABA’s suppression of photosynthesis and antioxidant systems by antagonizing the excessive accumulation of ABA signaling under salt stress. Furthermore, comprehensive reviews highlight that melatonin’s protection in plants often extends to the regulation of ionic homeostasis, such as maintaining K^+^/Na^+^ balance [15]. However, in stark contrast, the specific mechanisms of melatonin action in woody plants—including whether it relies on similar pathways or employs distinct strategies—remain poorly understood [16,17]. In particular, there is a lack of integrated studies dissecting how melatonin systemically coordinates the antioxidant system with transcriptional and metabolic reprogramming under salt stress in trees.

Given melatonin’s known capacity to regulate redox homeostasis and systemic acclimation [18,19], we hypothesize that exogenous melatonin enhances salt tolerance in *Fraxinus* primarily by activating endogenous defense systems, including bolstering the antioxidant capacity and protecting photosynthetic function [20,21]. To test this hypothesis, this study treated *F. mandshurica* seedlings with a specific salt concentration (e.g., 400 mM Na_2_CO_3_/NaHCO_3_)—an established approach for mechanistic studies [22]—to simulate moderate salt stress. This concentration was selected based on preliminary experiments, as it induces a reproducible, moderate stress phenotype suitable for mechanistic studies. We systematically measured growth, oxidative damage markers (MDA), and antioxidant enzyme activities under melatonin treatment. Furthermore, through transcriptomic analysis, we aimed to identify core gene modules and metabolic pathways regulated by melatonin, thereby constructing a mechanistic framework at physiological and molecular levels. This study seeks to elucidate the physiological and molecular mechanisms by which exogenous melatonin enhances salt tolerance in *Fraxinus*.

The findings will not only provide theoretical support for melatonin applications in woody plant stress resistance but also offer novel strategies for enhancing the adaptability of sensitive tree species to saline environments through biostimulation.

## 2. Results

### 2.1. Melatonin Enhances the Salt Tolerance of Fraxinus mandshuric

**Figure 1:**
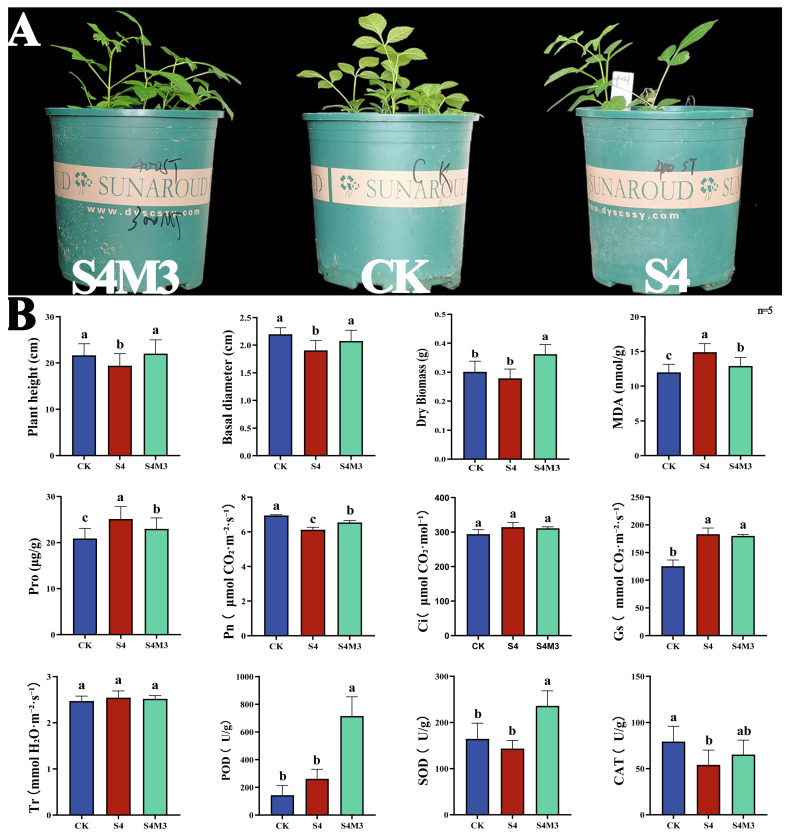
Melatonin alleviates saline-alkali stress induced growth inhibition and improves physiological performance in *Fraxinus mandshuric.* (**A**) Schematic representation of *F. mandshurica* growth under different treatments. (**B**) Growth and physiological metrics of *F. mandshurica* under different treatments. Pn, net photosynthetic rate; Ci, intercellular CO_2_ concentration; Gs, stomatal conductance; Tr, transpiration rate. Bars sharing the same lowercase letter are not significantly different, whereas those with different letters are significantly different.

Growth indicators showed that the melatonin group exhibited significantly higher seedling height, stem diameter, and dry biomass (whole plant) compared to the saline-alkali group. Shoot height increased by 13%, stem diameter by 8%, and dry biomass by 29% (Figure 1A). Concurrently, MDA and PRO con-tents in the melatonin group were significantly lower than in the saline-alkali group. The photosynthetic parameter Pn was also significantly higher in the melatonin group than in the saline-alkali group. While Ci and Tr showed no significant differences among groups, both melatonin and saline-alkali groups exhibited higher values than the control group. Regarding antioxidant enzymes, POD, SOD, and CAT levels were significantly higher in the melatonin group than in other groups (Figure 1B).

### 2.2. Transcriptomics and Functional Annotation of DEGs

**Figure 2:**
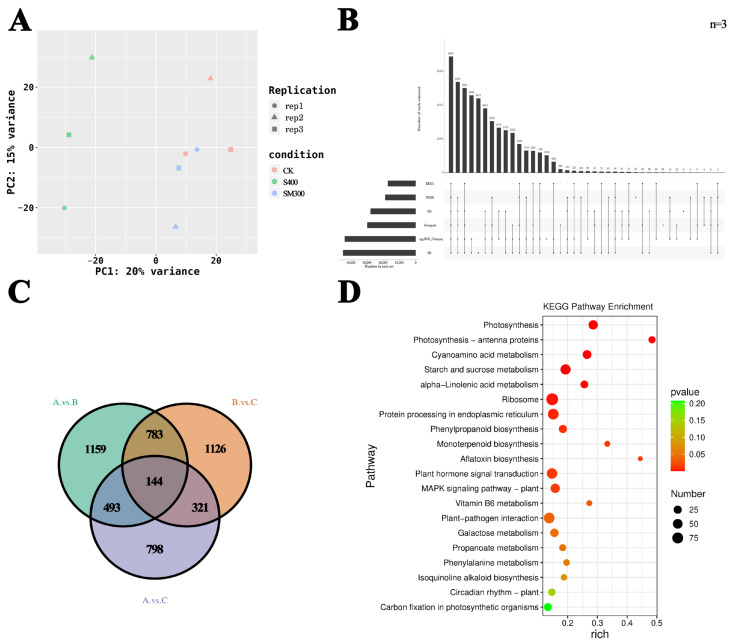
Transcriptomic analysis of *F. mandshurica*. (**A**) PCA results for the three treatments. (**B**) Annotation status of transcriptome sequencing. (**C**) Venn diagram depicting the distribution of DEGs among various treatments. (**D**) KEGG path-ways overrepresented in the various treatments.

Principal component analysis (PCA) plots reveal significant differences among the treatment groups, and consistent patterns are observed among three biological replicates within each group (Figure 2A). Transcripts from the transcriptome sequencing are annotated using several databases, including NR, GO, KEGG, Pfam, eggNOG, and Swiss-Prot, with successful annotation rates of 50%, 31%, 19%, 21%, 49%, and 33%, respectively (Figure 2B). In total, 4824 DEGs are identified across the three treatment groups. Specifically, 2579 DEGs are identified between the CK and S400 groups, 1756 DEGs between the CK and SM300 groups, and 2374 DEGs between the S400 and SM300 groups. There are 1159 distinct DEGs in the CK vs. S400 comparison, 798 distinct DEGs in the CK vs. SM300 comparison, and 1126 distinct DEGs in the S400 vs. SM300 comparison. Furthermore, the number of DEGs shared among all three groups is 144 (Figure 2C). KEGG enrichment analysis reveals several important pathways, including Plant hormone signal transduction, starch and sucrose metabolism, and phenylpropanoid biosynthesis (Figure 2D).

### 2.3. Metabolomics Analysis and Profiling of DAMs

**Figure 3:**
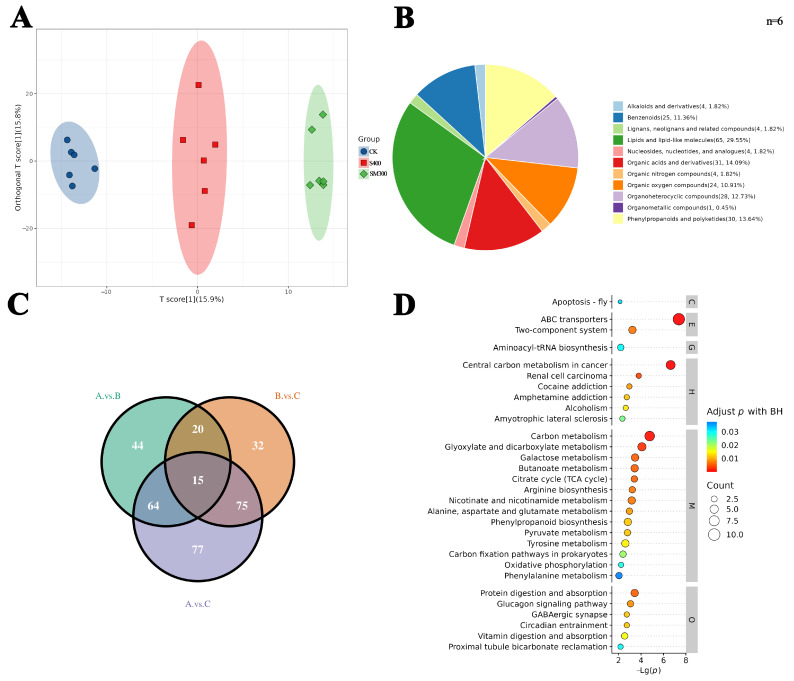
Metabolic analysis of *F. mandshurica*. (**A**) OPLS-DA analysis of the treatment groups. (**B**) Pie chart depicting the distribution of metabolite species. (**C**) Venn diagram showing the distribution of DAMs across different treatments. (**D**) KEGG pathways overrepresented in the various treatments, M, Metabolism; G, Genetic Information Processing; E, Environmental Information Processing; C, Cellular Processes; O, Organismal Systems; H, Human Diseases.

OPLS-DA analysis reveals significant differences among the various treatment groups, showing similar outcomes across all six replicates (Figure 3A). In total, 11 types of differential metabolites are identified across the groups, including lipids and lipid-like molecules, organic acids and derivatives, polyketides, phenylpropanoids and organic oxygen compounds, and benzenoids, which are the main classes of DAMs (Figure 3B). In total, 327 differential metabolites are identified across the three groups: 44 specific to the CK vs. S400 comparison, 77 unique to the CK vs. SM300 comparison, 32 exclusive to the S400 vs. SM300 comparison, and 15 common to all three groups (Figure 3C). KEGG enrichment analysis reveals several important pathways, including Circadian entrainment and Phenylpropanoid biosynthesis (Figure 3D).

### 2.4. Association Between Transcriptomics and Metabolomics

**Figure 4:**
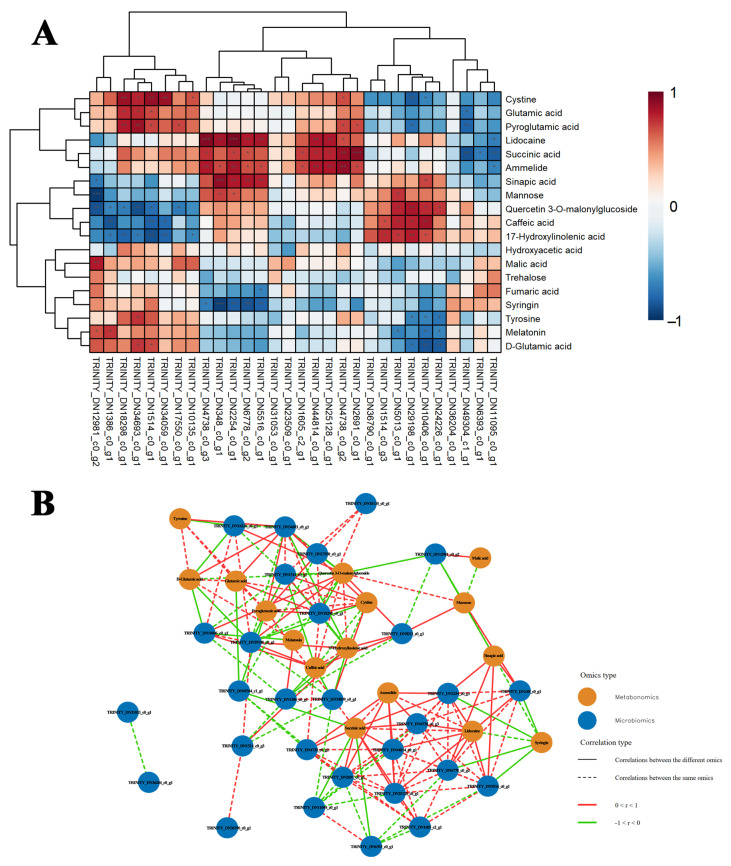
Integrated multi-omics analysis reveals coregulated genes and metabolites. (**A**) Heatmap of correlation between DEGs and DAMs, with red representing positive correlations and blue representing negative correlations, Asterisks indicate statistical significance: * *p* < 0.05, ** *p* < 0.01, *** *p* < 0.001. (**B**) Network of DEGs and DAMs. Yellow circles indicate metabolites; blue circles indicate genes. Solid lines indicate gene-to-gene or metabolite-to-metabolite relationships, while dashed lines represent gene-to-metabolite relationships. Red lines denote positive relationships, while green lines signify negative relationships.

The selected pathways include plant hormone signal transduction, circadian rhythm (plant), starch and sucrose metabolism and phenylpropanoid biosynthesis. Additionally, the study highlights DEGs and DAMs that exhibited the highest absolute correlation coefficients across the two omics datasets (Figure 4A). We also construct an interaction network between DEGs and DAMs within the key pathways mentioned above to illustrate the close relationship between gene expression and metabolite accumulation. This approach aims to elucidate the impact of gene regulation on metabolic pathways and deepen understanding of the underlying biological processes (Figure 4B, Table S1).

### 2.5. Circadian Rhythm—Plant and Plant Hormone Signal Transduction

**Figure 5:**
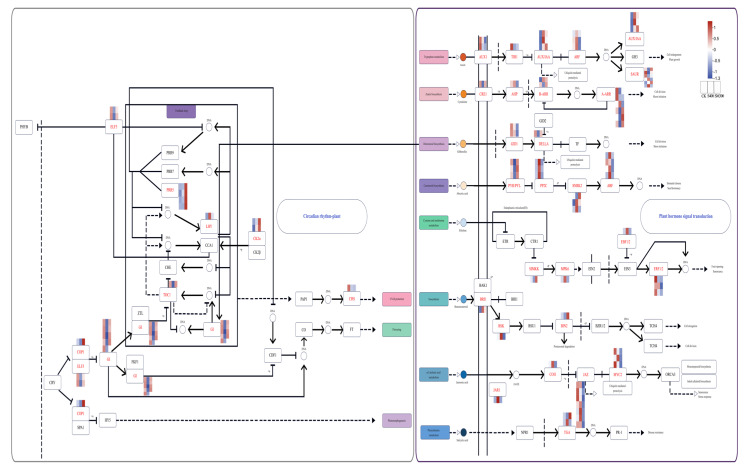
Melatonin modulates plant hormone signaling pathways. The diagram of the Circadian rhythm (*p* = 0.14)—plant and plant hormone signal transduction pathway (*p* = 0.012). Gray borders indicate *p*-values > 0.05, heatmap of correlation between DEGs, where red indicates positive associations and blue indicates negative associations.

In addition, 64 DEGs are identified within the plant hormone signal transduction pathway. Notably, *AUX1* and *TIR1* in the IAA pathway exhibit high expression in the CK group. In the CTK pathway, *CRE1*, *B-ARR*, and *A-ARR* are highly expressed in the SM300 group; in contrast, in the GA pathway, *DID1* is highly expressed in the S400 group, and *DEELA* shows higher expression in the SM300 group. In the ABA pathway, *PYR/PYL* are highly expressed in the SM300 group, while *PP2C* is prominently expressed in the S400 group, exhibiting differential expression between the SM300 and S400 groups, suggesting that the treatments significantly impacted distinct phytohormone signaling pathways. Notably, although not reaching statistical significance (*p* = 0.14), pathways associated with circadian rhythms also showed a certain degree of enrichment trend. This suggests that the mechanism by which exogenous melatonin alleviates salt stress may extend beyond classical hormone signaling pathways and potentially involve regulation of the plant’s intrinsic circadian clock system. Furthermore, the melatonin pathway exhibited significant expression in this metabolome, a hypothesis that warrants validation through more refined temporal experiments in the future (Figure 5).

We establish a protein–protein interaction (PPI) network derived from the DEGs in the circadian rhythm-plant and plant hormone signal transduction pathways (Figure S1). E3 ubiquitin-protein ligase RFWD2 (COP1) and auxin influx carrier (AUX1) are recognized as central nodes in the network, implying that these genes are essential for regulating circadian rhythms and hormone signaling. These genes may contribute to the growth differences observed across the various treatment groups in *F. mandshurica*.

### 2.6. Carbon Fixation Pathway and Starch and Sucrose Metabolism in Carbon Metabolism

**Figure 6:**
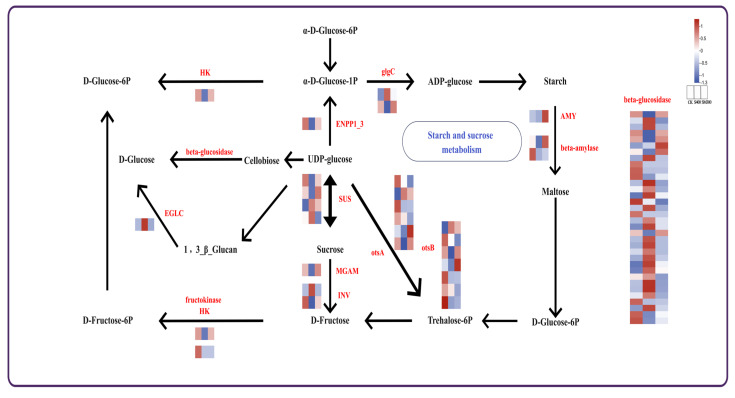
Starch and sucrose metabolism in carbon metabolism. Heatmap of correlation between DEGs and DAMs, where red indicates positive associations and blue indicates negative associations (*p* < 0.001).

Starch and sucrose metabolism are core components of photosynthesis in plants, serving essential functions in material accumulation and energy conversion that are vital for plant growth and development (Figure 6). In the starch and sucrose metabolism pathway, 60 DEGs are identified. *HK*, *ENPPI_3*, *MGAM* and *AMY* were upregulated in the SM300 group compared to the S400 group. Conversely, *EGLC* was significantly expressed in the S400 group. Additionally, *otsA* and *otsB* exhibit complex regulatory patterns.

We establish a protein–protein interaction (PPI) network derived from the DEGs in the starch and sucrose metabolism, as shown in (Figure S2). Beta-glucosidase [E3.2.1.21] emerges as a key hub in the network, suggesting that these genes play vital roles in starch and sucrose metabolism. These genes are likely crucial for material accumulation and energy metabolism.

### 2.7. Role of Phenylpropanoid Biosynthesis Pathways in Stress Tolerance

**Figure 7:**
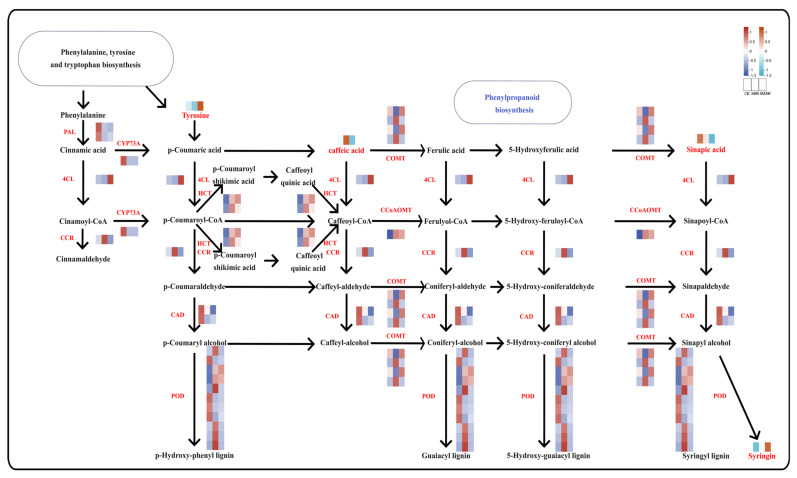
Role of phenylpropanoid biosynthesis pathways in stress tolerance (*p* < 0.01). Heatmap of correlation between DEGs and DAMs, where red indicates positive associations and blue indicates negative associations.

The phenylpropanoid pathway is a vital secondary metabolic route in plants, which significantly influenced plant growth, development, stress tolerance, and ecological adaptation (Figure 7). In our study, we identify 34 DEGs and 4 DAMs, including tyrosine, caffeic acid, sinapic acid, and syringin, along the phenylpropanoid pathways. Notably, tyrosine and syringin are highly accumulated in the SM300 group, whereas sinapic acid and caffeic acid are upregulated in the CK and S400 groups, respectively. Regarding the DEGs, *CCoAOMT* is upregulated in both the SM300 and S400 groups, while *4CL* and *COMT* are upregulated in the SM300 group, and *CCR* is significantly upregulated in the S400 group. The accumulation of sinapic acid is positively correlated with *CAD* expression, whereas syringin levels are negatively correlated with both *CAD* expression and sinapic acid concentration. Transcriptomic and metabolomic associations reveal that the fluctuations of these DAMs are tightly coordinated with the corresponding genes.

A protein–protein interaction (PPI) network is constructed using the DEGs identified in the phenylpropanoid biosynthesis pathways (Figure S3). In this network, caffeoyl-CoA O-methyltransferase (OMT1) serves as a gene hub, indicating that these genes could be pivotal in plant stress tolerance and ecological adaptation.

### 2.8. Mantel Test

**Figure 8:**
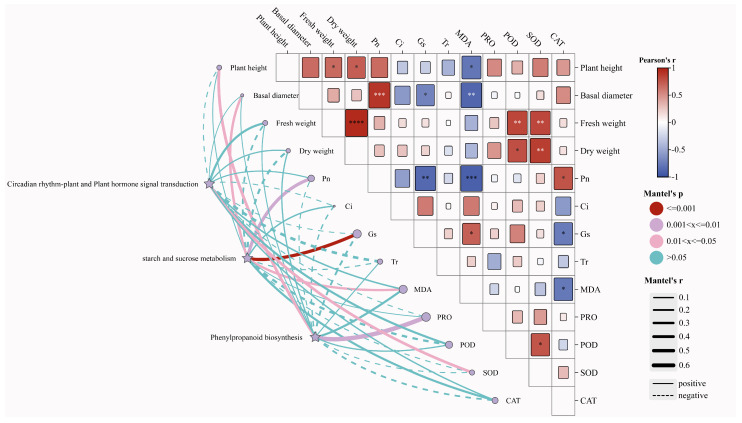
Mantel test correlates pathway activity with physiological outcomes: The diagram illustrates the relationship between the three core pathways and various growth and physiological indices. The size of the circle reflects the degree of influence of each pathway on the indices, with larger circles indicating a higher level of impact. The color of the connecting lines represents the significance of the relationships; meanwhile, the line thickness reflects the correlation strength. Thicker lines represent stronger correlations, with solid lines indicating positive relationships and dashed lines representing negative ones, Asterisks indicate statistical significance: * *p* < 0.05, ** *p* < 0.01, *** *p* < 0.001, **** *p* < 0.0001.

Plant height is positively correlated with Pn, PRO, POD, SOD, and CAT and negatively correlated with MDA and Gs. Basal diameter exhibits a strong positive correlation with Pn and a notable negative correlation with MDA and Gs. Dry Biomass exhibits a significant positive correlation with POD and SOD. These key pathways have a substantial impact on various indicators, including plant height, Dry biomass, Pn, Gs, MDA, PRO, POD, SOD, and CAT (Figure 8).

## 3. Discussion

This study demonstrates that exogenous melatonin confers salt tolerance to *Fraxinus mandshuric* by reshaping its carbon allocation strategy and stress signaling networks, rather than directly enhancing photosynthetic productivity. This reveals a distinct energy-saving stress response pattern mediated by melatonin in woody plants. This conclusion is supported by multi-level evidence: At the physiological level, melatonin treatment maintained high net photosynthetic rates without significantly altering stomatal conductance. At the transcriptomic level, pathways associated with carbon fixation remained inactive, while starch and sucrose metabolism pathways—responsible for carbon source mobilization and redistribution—were significantly enriched (*p* < 0.01). This indicates that melatonin guides plants to prioritize limited resources toward defense construction rather than attempting to enhance primary productivity already suppressed by stress.

This resource-optimization strategy contrasts with the more direct mechanisms often reported in herbaceous models. In plants such as tomato, cucumber, and rice, melatonin primarily enhances salt tolerance by mitigating immediate physiological injuries—scavenging reactive oxygen species (ROS) and rectifying hormone (e.g., ABA) signaling to restore homeostasis [12,13,14]. For the perennial woody species *F. mandshurica,* however, our data suggest a mechanism adapted to its long-term life strategy: instead of merely repairing acute damage, melatonin orchestrates a strategic re-allocation of internal resources (e.g., carbon) to prioritize sustained defense and maintenance over maximal growth, aligning with the ecological demands of a tree.

### 3.1. Coordinated Protection of Photosynthesis

In this study, saline-alkali stress significantly inhibited the net photosynthetic rate (Pn) but, unexpectedly, increased both stomatal conductance (Gs) and intercellular CO_2_ concentration (Ci). This specific pattern—decreased Pn coupled with increased Ci—clearly indicates that the inhibition of photosynthesis was primarily governed by non-stomatal limitations, i.e., direct damage to chloroplast structure and key photosynthetic enzymes (e.g., *Rubisco*) by saline-alkali ions, rather than by CO_2_ substrate deficiency [23,24,25]. The abnormally high Gs under stress may reflect a disruption in ABA signaling, leading to stomatal regulatory dysfunction [26,27]. Notably, the transpiration rate (Tr), which is primarily driven by stomatal aperture (Gs), consequently showed a similar pattern of increase under stress. This further indicates that melatonin did not enhance tolerance by suppressing transpiration for water conservation. Instead, it likely maintained water balance through alternative mechanisms, such as enhancing root water uptake or adjusting osmotic substances. Exogenous melatonin treatment effectively restored Pn without significantly altering Gs or Ci. This strongly suggests that melatonin’s protective action targets the photosynthetic apparatus downstream of the stomata. Concurrently, melatonin treatment significantly enhanced the plant’s antioxidant defense system: the activities of peroxidase (POD), superoxide dismutase (SOD), and catalase (CAT) were all significantly higher in the melatonin-treated group than in other groups [28]. Correspondingly, the contents of the oxidative damage marker malondialdehyde (MDA) and the osmoregulatory substance proline (PRO) were significantly lower in the melatonin group than in the saline-alkali stress group [29]. Therefore, we propose that melatonin likely alleviates photosynthetic decline by mitigating ROS-induced photoinhibition of photosystem II (PSII), protecting the electron transport chain, and potentially stabilizing Calvin cycle-related enzymes, thereby improving the leaf mesophyll’s capacity to utilize the available CO_2_ pool [30,31]. This synergistic effect—repairing internal photosynthetic efficiency while reinforcing the antioxidant barrier—ensures the continuity of carbon assimilation, which directly underpins the 29% increase in whole-plant dry biomass observed in the melatonin-treated group [32]. This mechanism of “repairing the internal factory efficiency and reinforcing its defenses, rather than merely widening the raw material import” constitutes a key characteristic of melatonin as an efficient biostimulant.

### 3.2. Energy Redistribution Strategy

First, as shown above, in the carbon metabolic cycle under melatonin treatment, the carbon fixation pathway was not upregulated (*p* = 0.2), while starch and sucrose metabolism were strongly activated. Simultaneously, *Fraxinus* failed to increase its energy supply (photosynthesis) under stress conditions, instead prioritizing conservation and redistribution. This resource re-allocation strategy under stress, shifting investment from growth to defense, is a conserved adaptive mechanism in plants [33]. Melatonin likely promotes the conversion of stored carbon (starch) into stress-responsive compounds (e.g., osmoregulatory substances, antioxidant substrates). Under energy-limited stress, successful adaptation often involves optimizing existing resource utilization rather than blindly increasing production [34,35]. This metabolic reprogramming provides a direct carbon skeleton and energy source for the observed massive accumulation of proline. Concurrently, this coordinated response is likely initiated by the upregulation of hormone signaling pathways (e.g., ABA, JA), which are known master regulators of defense resource allocation (*p* = 0.01 pathway) [36].

### 3.3. Synergy and Trade-Offs in Signaling Networks

Given that melatonin itself is a core circadian rhythm regulatory molecule, its exogenous application may leverage or mimic this endogenous regulatory function, revealing significant biological insights [37]. The mechanism by which exogenous melatonin alleviates saline-alkali stress likely extends beyond classical hormone signaling pathways and may involve potential interactions with the regulation of plants’ intrinsic circadian clock systems. Although the ‘circadian rhythm-plant’ pathway did not reach statistical significance, its enrichment trend suggests an intriguing possibility: melatonin, as a core timing molecule, may fine-tune the plant’s intrinsic clock through exogenous application, thereby optimizing circadian-dependent stress responses (e.g., stomatal opening/closing, rhythmic antioxidant enzyme activity) [38,39,40]. This could relate to the observed reduction in oxidative damage. Future studies should validate this hypothesis at finer temporal resolutions.

### 3.4. Building a Core Defense Arsenal

Transcriptome analysis indicates that the phenylpropanoid biosynthesis pathway serves as a central downstream target regulated by melatonin. Activation of this pathway directly channels carbon flux toward the production of multiple key stress-resistant compounds. The phenylpropanoid pathway is the sole synthetic source for phenolic compounds such as plant flavonoids and lignin monomers. These compounds serve as potent non-enzymatic antioxidants capable of directly scavenging excess reactive oxygen species [41,42]. The significant reduction in oxidative damage markers (MDA) observed in melatonin-treated groups likely stems directly from pathway activation, which replenishes endogenous antioxidant reserves. This compensates for potential limitations of relying solely on enzymatic systems like SOD and POD, forming a more robust antioxidant defense system. Moreover, the pathway’s end product, lignin, serves as a key substrate for cell wall lignification [43,44]. Under salt stress, lignin deposition strengthens cell walls and reduces their permeability. This not only helps maintain cell structure and resist wilting caused by osmotic stress but may also physically restrict rapid Na^+^ transport to the aboveground parts via the apoplast pathway. The activation of this pathway in *Fraxinus* in response to melatonin parallels findings in *Solanum lycopersicum*, where melatonin also induces phenylpropanoid-related genes under salinity stress, suggesting a potentially conserved mechanism across species [45], highlighting the potential generality of this mechanism. Our study extends this observation to a perennial woody species, confirming its relevance in forest trees. Thus, activation of the phenylpropanoid pathway serves as the biochemical hub through which melatonin implements a dual-pronged strategy of oxidative protection and physical reinforcement.

It should be noted that the conclusions of this study are based on pot experiments during the seedling stage and specific stress intensities/durations. Plant stress responses are indeed highly dependent on developmental stage and environmental conditions [46]. Therefore, our findings primarily reveal melatonin’s core role in the initial phase of acute salt stress response in *F. mandshurica* seedlings. Whether this ‘energy-conserving’ defense strategy remains dominant under prolonged field stress or in mature trees warrants further investigation.

**Figure 9:**
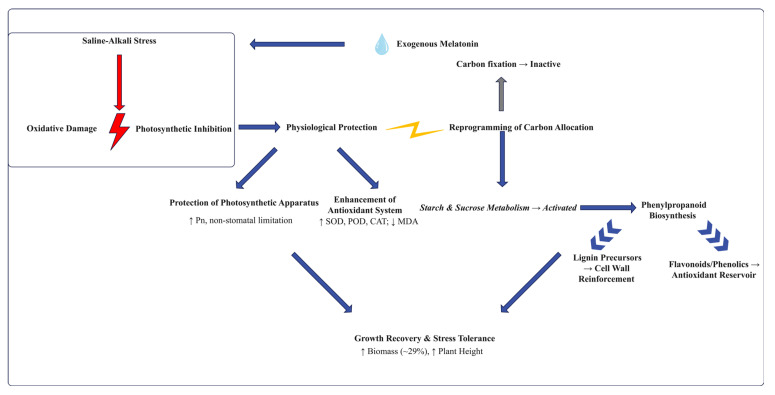
A proposed model for melatonin-mediated saline-alkali tolerance in *F. mandshurica*. Exogenous melatonin alleviates stress by (1) protecting the photosynthetic apparatus and (2) enhancing the antioxidant enzyme system. Concurrently, it (3) reprograms central carbon metabolism, redirecting flux from starch/sucrose metabolism into the phenylpropanoid biosynthesis pathway, which produces compounds for lignin deposition (physical reinforcement) and antioxidant phenolics (chemical defense). These coordinated responses collectively lead to improved growth and stress tolerance. In this schematic, red indicates stress/impairment, blue indicates melatonin treatment/protective effects, and gray indicates inactive pathways. Solid arrows indicate pathways strongly supported by the data; dynamic arrows indicate putative relationships, Upward arrows (↑) denote increase or upregulation, and downward arrows (↓) denote decrease or downregulation.

In summary, exogenous melatonin initiates early hormonal signaling, strategically redirecting carbon resources from storage (starch) to defense metabolism, primarily via the phenylpropanoid biosynthesis pathway. This process efficiently synthesizes multiple stress-resistant compounds, including lignin precursors and flavonoids, without significantly increasing the burden on primary photosynthetic capacity. Consequently, it synergistically enhances oxidative scavenging, cell wall reinforcement, and ion compartmentalization, systematically improving the salt tolerance of *F. mandshurica*. This integrated energy-efficient defense strategy is summarized in our proposed mechanistic model (Figure 9). The model illustrates how melatonin orchestrates protection of the photosynthetic apparatus, enhancement of the antioxidant system, and reprogramming of carbon allocation toward defense compound biosynthesis, collectively leading to growth recovery. These findings provide new insights into melatonin’s function as a systemic defense regulator in woody plants. Future work targeting key phenylpropanoid pathway genes via gene editing, combined with field trials, will advance melatonin from a promising candidate molecule to practical applications in forestry stress resistance.

## 4. Materials and Methods

### 4.1. Plant Materials

Seeds were sourced from Jitai Nursery Company, Yichun City, Heilongjiang Province, China. Experiments were conducted in the artificial climate chamber at Jilin Agricultural University under the following conditions: indoor temperature 26 ± 2 °C, indoor humidity 50–70%, light intensity 2000 lux, and light cycle 14/8 h. Watering was performed every two days to maintain humidity. Whole, plump seeds were surface-disinfected by soaking in a 1% sodium hypochlorite solution for 15 min [47]. Seeds were rinsed five times with de-ionized water, then soaked in de-ionized water for 24 h before being placed in seedling trays. The soil substrate in the trays consisted of peat moss/perlite/vermiculite in a 2:1:1 ratio. After one month of growth, seedlings were thinned to retain uniformly developed, vigorous plants.

### 4.2. Experiment Treatment

To implement stress treatment, uniformly developed one-year-old *F. mandshurica* seedlings were selected. Based on preliminary experimental results, the experimental groups were divided into three treatments: a control group (CK) with normal watering; an alkali salt stress group (S400) treated with a 400 mmol/L solution of Na_2_CO_3_ and NaHCO_3_ mixed in equal proportions; and a melatonin-alkaline salt stress group (SM300), where plants received the alkaline salt treatment followed by spraying with a 300 μmol/L until runoff (~10 mL per plant) at dusk. Melatonin solution preparation: Weigh 0.232 g melatonin (McLean) and dissolve in 5 mL anhydrous ethanol. Dilute with deionized water to 250 mL to prepare a 4 mM stock solution. Prior to use, further dilute the stock solution with de-ionized water containing 0.05% (*v*/*v*) Tween-20 to prepare a 300 μM working solution. Before formal stress application, all potted seedlings underwent uniform water restriction pretreatment to establish consistent initial water content conditions. Procedure: Plants were thoroughly watered prior to pretreatment, followed by cessation of water supply. Soil moisture loss was monitored via daily weighing. When pot weight reached the target value (equivalent to 75% of maximum water-holding capacity), salt stress or control treatment was initiated for that pot. At the start of stress treatment, all treated pots received a single irrigation with 400 mM Na_2_CO_3_/NaHCO_3_ solution to adjust soil moisture content to 75% field capacity. Control pots reached equivalent moisture content with an equal volume of deionized water. Thereafter, evaporation losses were replenished every two days via weighing, and corresponding solutions were applied to maintain soil moisture at 75%. Melatonin solution was sprayed at dusk on days 0, 3, and 7 following salt solution application. Leaf samples were collected from all treatment groups starting on day 30 for subsequent analysis. Growth indices and physiological measurements were assessed using 5 biological replicates. Transcriptome analysis included 3 biological replicates, while metabolome analysis comprised 6 biological replicates.

### 4.3. Determination of Growth and Physiological Indicators

Plant height and basal diameter were measured using a straightedge and basal diameter. For dry weight determination, samples were first transferred to an oven heated to 105 °C for 30 min to blanch the tissue, followed by drying at 75 °C until constant weight was achieved. Biomass dry weight was recorded using a 1/10,000 precision electronic balance [47]. Photosynthetic parameters were obtained from fully expanded leaves located at the top of *F. mandshurica* seedlings between 9:00 and 11:00 a.m. using a portable photosynthesizer (CIRAS-3, USA) [48]. The concentrations of malondialdehyde (MDA) [49], proline (PRO) [50], peroxidase (POD) [51], superoxide dismutase (SOD) [52], and catalase (CAT) [53] in plant tissues were measured using an ELISA kit from Suzhou Geruisi Biotechnology Co., Ltd. (Suzhou, China).

### 4.4. RNA Extraction

Total RNA was extracted and mRNA was enriched using Oligo(dT) magnetic beads. Sequencing libraries were constructed following the standard Illumina protocol: RNA fragmentation (~300 bp), cDNA synthesis with random primers, and PCR amplification. Library quality and concentration were assessed (Agilent 2100 Bioanalyzer) before pooling.

### 4.5. Transcriptome Sequencing

Following library construction and quality assessment (Agilent 2100 Bioanalyzer), pooled libraries were sequenced on an Illumina NovaSeq 6000 platform to generate paired-end reads [54]. Raw reads were quality-filtered to remove adapters, low-quality sequences (Q20), and short reads (<50 bp). High-quality reads were de novo assembled into transcripts and clustered to generate a unigene set. These unigenes were functionally annotated against multiple databases (GO, KEGG, eggNOG, Swiss-Prot, Pfam). Clean reads were mapped back to unigenes to obtain read counts. Differential gene expression analysis was performed using DESeq2 [55], and KEGG pathway enrichment analysis was conducted with ClusterProfiler [56].

### 4.6. Metabolite Sample Preparation

Fresh samples were weighed, lyophilized, and homogenized (Grinding Mill, 65 Hz, 1 min) in tubes with a tungsten bead. Metabolites were extracted with 1 mL of precooled methanol/acetonitrile/water (2:2:1, *v*/*v*/*v*) by ultrasonication on ice for 1 h. The extracts were incubated at −20 °C for 1 h, then centrifuged (14,000× *g*, 20 min, 4 °C), and the supernatants were vacuum-dried. Profiling was performed using a UPLC-ESI-Q-Orbitrap-MS system (Shimadzu Nexera X2 coupled to a Q Exactive Plus, Thermo Scientific, Waltham, MA, USA).

### 4.7. UHPLC-MS/MS Analysis

Chromatographic separation was performed on an ACQUITY UPLC HSS T3 column (2.1 × 100 mm, 1.8 μm; Waters) at 0.3 mL/min using a gradient of 0.1% formic acid in water (A) and acetonitrile (B). Mass spectrometry data were acquired in both positive and negative ESI modes on a Q Exactive Plus instrument. Full MS scans (*m*/*z* 70–1050) were obtained at a resolution of 70,000, with data-dependent MS/MS scans at 17,500 a resolution.

Raw data were processed using MS-DIAL for peak picking, alignment, and annotation. Metabolites were identified by matching accurate mass (mass error < 10 ppm) and MS/MS spectra (mass error < 0.02 Da) against public databases (HMDB, MassBank) and a custom standard library [57,58]. For data filtering, features were first retained only if they were detected in over 50% of the samples within at least one experimental group. Subsequently, an additional quality control step was applied: only features with a relative standard deviation (RSD) < 30% in the pooled quality control (QC) samples were retained for subsequent analysis.

### 4.8. Integrated Metabolomic and Transcriptomic Analysis

The Pearson correlation coefficient was used to evaluate the associations between differentially expressed genes (DEGs) identified from transcriptomics and differentially abundant metabolites (DAMs) identified from metabolomics. A correlation coefficient less than 0 indicated a negative relationship, while a coefficient greater than 0 signified a positive relationship.

### 4.9. Analysis of Protein–Protein Interaction Networks and Identification of Hub Genes

The protein–protein interaction (PPI) network was built using STRING (https://string-db.org/ (accessed on 8 June 2025)), following the method outlined [59]. Hub nodes were determined as those exhibiting the most frequent interactions with adjacent nodes. The resulting data were then imported into Cytoscape software (v3.9.0), where Betweenness Centrality analysis was conducted to pinpoint key hubs within the network.

### 4.10. Real-Time PCR Analysis

The expression levels of the chosen differentially expressed genes were assessed via RT-qPCR, with 3 biological replicates to validate the reliability and stability of the transcriptomic data. The SYBR Green fluorescence-based quantitative method was employed to measure and confirm the expression of 6 target genes. Relative gene expression levels were calculated using the 2^(−ΔΔCt) method [60], with Fm-TU acting as the reference gene.

### 4.11. Data Evaluation

Growth indicators and physiological measurements were analyzed using five biological replicates, transcriptomics with three biological replicates, and metabolomics with six biological replicates. IBM SPSS Statistics 26.0 software was employed for chi-square tests, analysis of variance (ANOVA), and normality tests, with significant differences determined using the LSD method. Bar charts for growth and physiological indicators were generated using GraphPad Prism 10.1.2 software. Correlation heatmaps were constructed using Pearson’s correlation coefficient (r). Matrix relationships among genes, metabolites, and physiological factors in transcriptomics and metabolomics were analyzed via Mental Test. Sequencing services were provided by Shanghai BioPro Bio-Tech Co., Ltd. (Shanghai, China).

## 5. Conclusions

This study demonstrates that exogenous melatonin alleviates combined saline-alkali stress in *Fraxinus mandshurica* seedlings. Crucially, melatonin conferred tolerance not by boosting overall metabolism, but by orchestrating a strategic reallocation of internal resources—a distinct energy-saving response pattern in this woody species. This was evidenced by (1) improved growth (increased biomass) alongside protected photosynthesis, (2) enhanced antioxidant capacity (elevated POD/SOD, decreased MDA), and (3) the specific reprogramming of defense metabolism, particularly the activation of the phenylpropanoid biosynthesis pathway. Therefore, melatonin enhances resilience primarily by optimizing carbon allocation toward defense rather than growth, underpinned by a reconfigured transcriptomic and metabolic network. This work provides an empirical basis for applying melatonin to improve tree adaptability in saline environments.

## Data Availability

Data will be made availability on request.

## Supplementary Materials

The following supporting information can be downloaded at https://www.mdpi.com/article/10.3390/plants15030438/s1, Figure S1: PPI network of DEGs identified in the Circadian rhythm and plant hormone signal transduction pathways; Figure S2: PPI network of DEGs in the Starch and Sucrose Metabolism pathways; Figure S3: PPI network of DEGs identified in the phenylpropanoid biosynthesis pathways; Figure S4: Relative expression trends in DEGs verified by RT-qPCR. Table S1: DEGs and DAMs network; Table S2: qRT-PCR primer sequence information.
