# Supplementary material for: Exogenous Melatonin Confers Salt-Alkali Tolerance in Fraxinus mandshurica by Orchestrating Resource Allocation and Activating Phenylpropanoid-Mediated Defenses
Source: Plants (Basel). 2026 Jan 30;15(3):438. doi: 10.3390/plants15030438 (PMC12899434; doi:10.3390/plants15030438)
Supplement: Supplementary file 1 [file plants-15-00438-s001.zip › Appendix of Results.pdf]

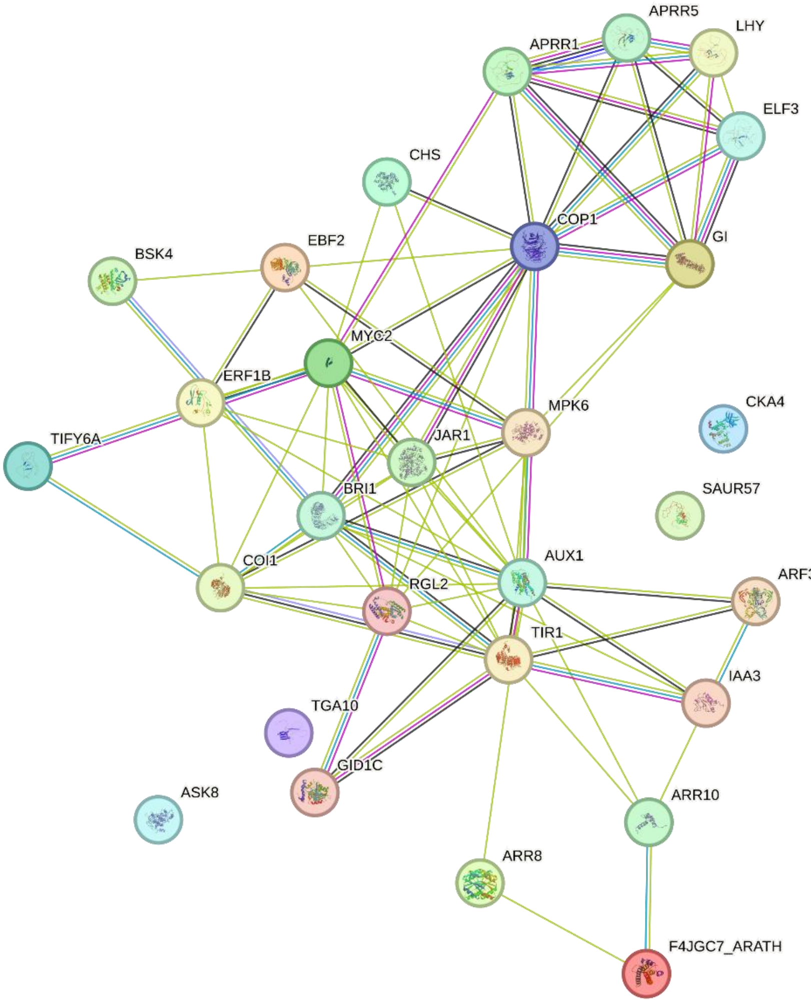

Figure.S1 PPI network of DEGs identified in the Circadian rhythm and plant hormone signal transduction pathways

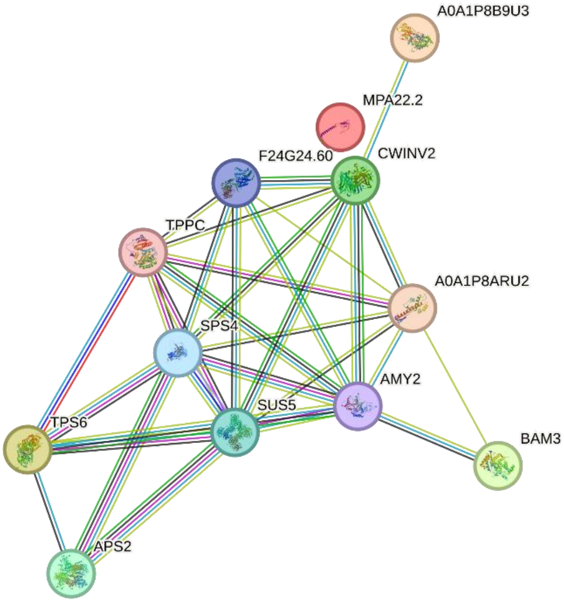

Figure.S2. PPI network of DEGs in the Starch and Sucrose Metabolism pathways.

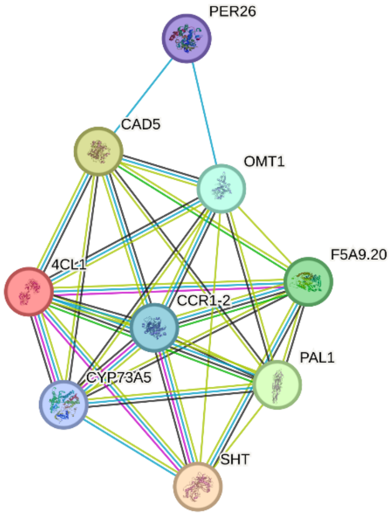

Figure.S3. PPI network of DEGs identified in the phenylpropanoid biosynthesis pathways.

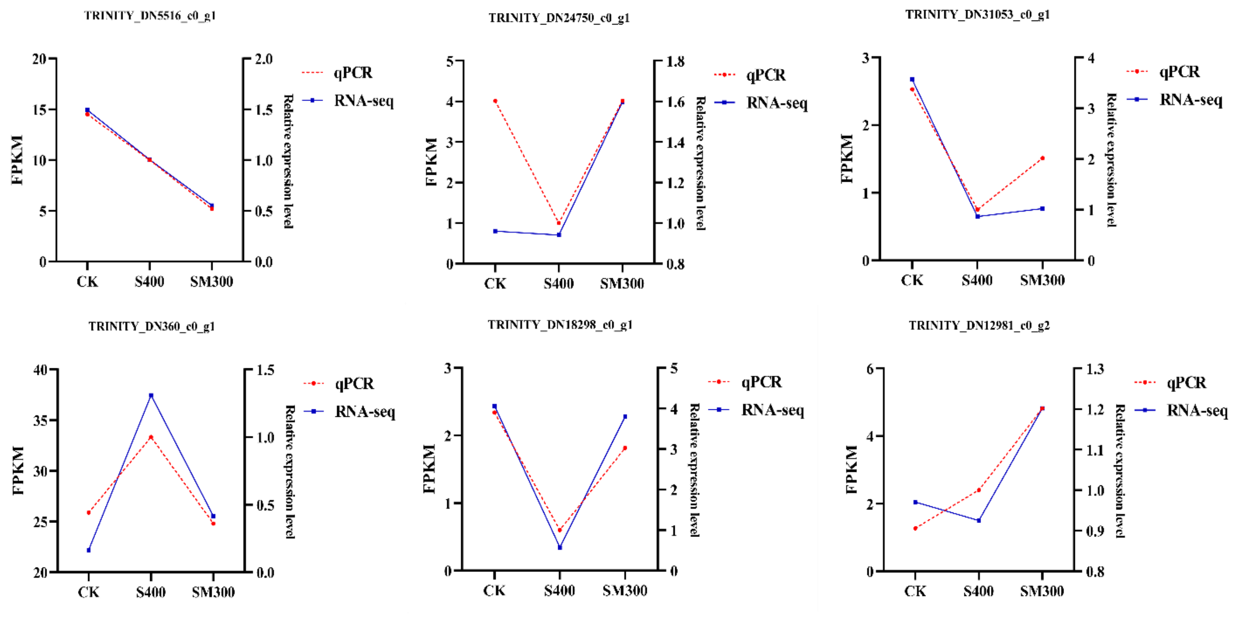

Figure.S4. Relative expression trends in DEGs verified by RT-qPCR. The concordant trajectory of the two lines for each gene provides direct visual evidence of the high correlation between the two independent methodological measurements, thereby confirming the reliability of the transcriptome sequencing data.
